# Swallowing interventions for the treatment of dysphagia after head and neck cancer: a systematic review of behavioural strategies used to promote patient adherence to swallowing exercises

**DOI:** 10.1186/s12885-016-2990-x

**Published:** 2017-01-10

**Authors:** Roganie Govender, Christina H. Smith, Stuart A. Taylor, Helen Barratt, Benjamin Gardner

**Affiliations:** 1 University College London, Health Behaviour Research Centre & University College London Hospital, Head & Neck Cancer Centre, Ground Floor Central, 250 Euston Road, London, NW1 2PQ UK; 2 Division of Psychology & Language Sciences University College London, London, UK; 3 Centre for Medical Imaging, University College London, London, UK; 4 Department of Applied Health Research, University College London, London, UK; 5 Department of Psychology, Institute of Psychiatry, Psychology and Neuroscience (IoPPN), Kings College London, London, UK & UCL Department of Epidemiology & Public Health, University College London, London, UK

**Keywords:** Dysphagia, Head neck cancer, Swallowing exercises, Behavior change techniques, Adherence, Complex interventions

## Abstract

**Background:**

Dysphagia is a significant side-effect following treatment for head and neck cancers, yet poor adherence to swallowing exercises is frequently reported in intervention studies. Behaviour change techniques (BCTs) can be used to improve adherence, but no review to date has described the techniques or indicated which may be more associated with improved swallowing outcomes.

**Methods:**

A systematic review was conducted to identify behavioural strategies in swallowing interventions, and to explore any relationships between these strategies and intervention effects. Randomised and quasi-randomised studies of head and neck cancer patients were included. Behavioural interventions to improve swallowing were eligible provided a valid measure of swallowing function was reported. A validated and comprehensive list of 93 discrete BCTs was used to code interventions. Analysis was conducted *via* a structured synthesis approach.

**Results:**

Fifteen studies (8 randomised) were included, and 20 different BCTs were each identified in at least one intervention. The BCTs identified in almost all interventions were: *instruction on how to perform the behavior*, *setting behavioural goals* and *action planning*. The BCTs that occurred more frequently in effective interventions, were: *practical social support*, *behavioural practice*, *self*-*monitoring of behaviour* and *credible source* for example a skilled clinician delivering the intervention. The presence of identical BCTs in comparator groups may diminish effects.

**Conclusions:**

Swallowing interventions feature multiple components that may potentially impact outcomes. This review maps the behavioural components of reported interventions and provides a method to consistently describe these components going forward. Future work may seek to test the most effective BCTs, to inform optimisation of swallowing interventions.

**Electronic supplementary material:**

The online version of this article (doi:10.1186/s12885-016-2990-x) contains supplementary material, which is available to authorized users.

## Background

Swallowing difficulties (dysphagia), which affect 60 –75% of patients treated for head and neck cancer (HNC) [1], arise both from the presence of a tumour, and as a consequence of its treatment [2]. Dysphagia is a major patient concern after cancer treatment due to the detrimental impact on patients’ quality of life (QOL) [3]. Improvement of swallowing function and earlier restoration of eating and drinking after surgery or chemo-radiation treatments may be achieved with swallowing rehabilitation exercises [4, 5]. Despite this, non-adherence to swallowing exercises in this population is reported to be high [6].

The World Health Organization report defines patient adherence as “the extent to which a person’s behaviour corresponds with agreed recommendations from a health care provider” [7]. This report highlights that adherence is influenced by multiple factors, and that increasing adherence to treatment could have a greater impact on health than trying to improve the efficacy of the treatment to which patients are encouraged to adhere. Adopting this perspective transforms the concept of patient adherence from a peripheral marker of study quality into a concept central to the intervention. The Medical Research Council’s “complex intervention” guidelines highlight that multiple components at different levels may interact to bring about desired health outcomes [8]. Effectiveness of swallowing exercise interventions are determined not just by the exercises but also the broader ‘behaviours of those delivering and receiving the intervention’ (p.979). Complex interventions that take place as *pragmatic trials* under real-world conditions [9] are influenced by context factors; how interventions are implemented (where, by whom) and how patients may respond to this (uptake/adherence) [10].

Newer paradigms in systematic reviewing such as realist reviews focus on understanding how and why interventions work in some situations and not others, rather than simply investigating whether they do or do not work [11]. Sutcliffe and colleagues [12] argue the importance of recognising and identifying the critical components of complex interventions highlighting that outcomes of complex interventions cannot be solely ascribed to the primary content, in this case swallowing exercises. Traditional systematic reviews that focus exclusively on pooling effect sizes may overlook other aspects that influence outcomes. This limits our ability to differentially examine the evidence and to gather important information that may improve future interventions.

**Fig. 1:**
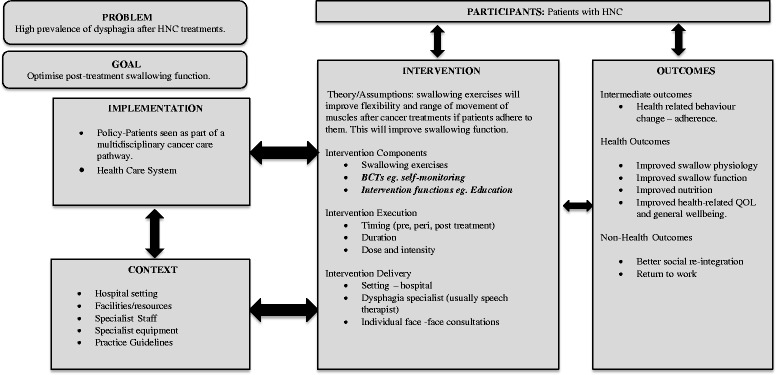
Logic Model of exercise interventions to improve swallowing in patients treated for head and neck cancer

The system in which the intervention takes place and the possible interactions that may occur can be represented as a logic model [13] (Fig. 1). Swallowing exercise interventions for patients with HNC are normally implemented by trained professionals such as speech therapists within a healthcare setting, and as part of a wider cancer care pathway. The content of the intervention tends to be focused on type, timing and intensity of different swallowing exercises. Accordingly, previous reviews have been largely concerned with these exercise parameters. Langmore and Pisegna [14] suggest that exercises such as the Shaker (head lift exercise) and Mendelsohn manoeuvre (larynx elevation exercise) have good efficacy in improving swallowing function. A general review of interventions to improve eating and drinking after HNC [15] concluded that some evidence exists to support exercises to improve swallowing function and jaw movement in patients treated for HNC but acknowledged that larger controlled studies are needed. A recent Cochrane review [16] concluded that the evidence for pre-treatment swallowing exercises in improving swallowing safety and efficiency is lacking due to insufficiently robust studies, heterogeneity of outcome measures across studies, and poor patient adherence. Whilst there is much to be learned from these reviews, the broader perspective proposed in our logic model may facilitate better understanding of the existing evidence that could improve the content and design of future studies (Fig. 1).

As highlighted in our model, behavioural strategies used to promote adherence to the exercises are an important *part of the intervention content* that may be frequently overlooked yet such strategies may have a potentially crucial influence on outcomes. This review employs established tools from Behavioural Science, in particular the Behaviour Change Technique Taxonomy (BCTTv1) [17] that defines 93 discrete behaviour change techniques (BCTs) thereby facilitating a standardised description of the techniques that can be used to change behaviour. BCTs represent the smallest observable and replicable components that may bring about a change in behaviour [17], and therefore may be potentially active ingredients in an intervention [18] The success of exercise interventions is dependent on good adherence. It is logical therefore that this aspect of the intervention be given appropriate consideration.

In this review, we aim to identify the specific behaviour change strategies reported in interventions to improve swallowing function after HNC. We also explored where possible, relationships between the presence of these components and intervention effectiveness. We propose that BCTs that occur at least twice as frequently in successful interventions may be useful to include in future interventions. We used a narrative synthesis approach [19] and as part of this we also explored the trial methods used more broadly (for example type of comparator group), providing discussion of possible associations with the study outcomes. To our knowledge this is the first attempt to apply this method of reviewing swallowing interventions within this field, and by its nature the work is exploratory.

### Methods

The review is registered with PROSPERO (CRD42015017048), and a protocol reporting full methodological detail has been published [20].

### Eligibility Criteria

Studies were eligible for inclusion where they met the following PICO criteria [21]. *Participants* were adults diagnosed with head and neck cancer; treated *via* one of the key treatment modalities of surgery, radiotherapy, chemo-radiotherapy or combinations thereof. *Interventions* that were eligible included behavioural interventions to improve swallowing such as swallowing exercises or instructions to adhere to a specific diet texture, and other specific swallowing strategies. Studies that included an independent *comparator* group were eligible - these could be randomised or non-randomised studies. The comparator group could have received no treatment (non-active comparator), usual care (active or non-active) or a different treatment (active) or sham exercise (active). For inclusion, the study had to report at least one swallow-related *outcome measure* which could be for example; swallow safety, swallow efficiency, swallow related QOL, oral diet intake or a surrogate marker such as feeding tube use, and textures of food tolerated. Evaluation could be *via* an established patient reported questionnaire, clinician rated measure or instrumental assessment tool such as videofluoroscopy.

### Identification of studies

Six electronic health databases were searched: Medline, CINAHL, EMBASE, AMED, PsychInfo, and the Cochrane Library including CENTRAL. Additional searches were carried out on Google Scholar, Web of Science and the meta-registries of Trials Databases (ClinicalTrials.gov and ISRCTN). Additionally, the WHO International Clinical Trials Registry Platform (ICTRP) and the Australian New Zealand Clinical Trials Register (ANZCTR) were searched. A hand-search of reference lists of directly relevant systematic reviews and included articles identified from the main screening was also undertaken.

The search strategy was developed in conjunction with a subject librarian, following an initial scoping exercise. Medical Subject Headings from key articles and other related reviews were examined to determine the final search terms. The search was limited to clinical trials and reviews published in English. No date limit was applied.

**Fig. 2:**
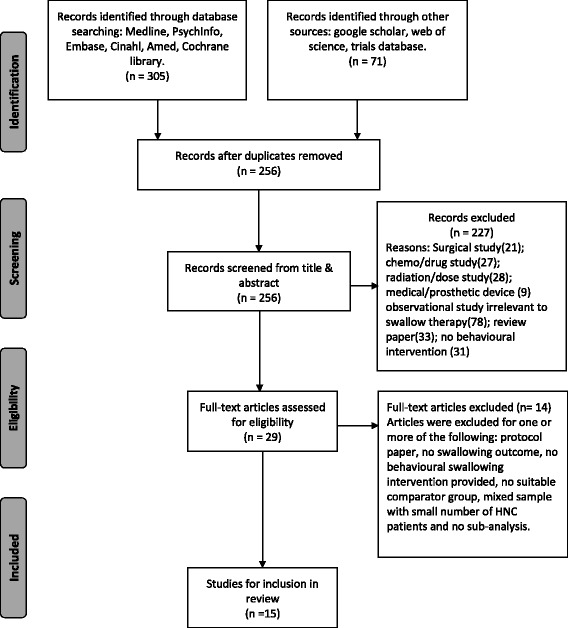
PRISMA flowchart showing process of study selection

Searches were carried out by a speech and language therapist (RG) and subject librarian (DG) in December 2014, and updated in June 2015 prior to completion of the data extraction process. One study [22] found to have two additional related reports based on longer follow-up times for the same sample and intervention, was treated as one study. Figure 2 depicts the PRISMA flowchart [23] showing the study selection process (Fig. 2).

### Data extraction

#### Study quality

For consistency with other reviews, data was extracted on study quality using an 11-item checklist [24] used previously to assess the quality of dysphagia clinical trials [25]. Each of the 11 items (Table 2) is given a score of 1 if the criterion is met, yielding a summary score of 0 (lowest) to 11 (highest quality). Van Tulder and colleagues [24] suggest that scores of ≥6 reflect studies of good quality. Studies were not excluded on the basis of quality because we aimed to ascertain any evidence, however weak, of potential links between BCTs and effects. Assessing study quality and potential risk of bias is still important when synthesizing findings even if only exploratory in nature [19].

#### Study characteristics

Data were extracted on study characteristics (author, year, country of origin, setting, type of study), patient characteristics (diagnostic and treatment group, sample size, age range, gender and baseline swallow function), treatment (information about the type of treatment and comparator groups), and outcome measures (length of follow-up and all swallow related outcomes). We anticipated heterogeneity in the type and time-points of outcome measures but an attempt was made to extract data at or as close to the time intervals of 1, 3, 6 and 12 months after treatment. They included measures derived from instrumental assessments such as modified barium swallow or videofluoroscopy, clinical measurements such as weight or the water swallow test (WST) [26], functional scales such as the Functional Oral Intake Scale (FOIS) [27] and Performance Status Scale (PSS) [28], patient-reported and QOL measures such as the MD Anderson Dysphagia Inventory (MDADI) [29] and European Organisation for Research and Treatment of Cancer (EORTC QOL C-30) [30] questionnaire.

#### Intervention Characteristics

For this review, we were particularly interested in identifying the behaviour change strategies (Additional file 1: Table S1 and Additional file 2: Table S2) present in the interventions. We recorded the target behaviour in each study, which was either regular performance of swallowing exercises or regular implementation of a prescribed diet modification with or without specific swallowing strategies. We intended to code for whether a named theory of behaviour or behaviour change was mentioned in the Abstract, Introduction, or Method, but no studies were found to have mentioned theory. We identified behaviour change strategies using BCTTv1. We also documented *Intervention Function* categories. Michie and colleagues [31] propose a list of nine Function categories that reflect the broad methods through which an intervention may influence behaviour: Education, Training, Enablement, Modeling, Restrictions, Environmental Restructuring, Persuasion, Incentivisation and Coercion. Both BCTs and intervention functions were only coded when they were unambiguously present in the intervention descriptions. For example if the intervention included a TheraBite device (Atos Medical, Sweden) to maintain mouth opening function – the intervention function *Education* was coded if it was clear that the intervention explicitly required that patients be informed and understand how the device and exercise works to maintain the ability to open the jaw. This may extend to information about the impact of radiotherapy on jaw movement and the consequences of doing/not doing the exercise. The function category *Training* was coded where it was clear that the patient was taught skills on how to perform the exercises using the device. The BCT *demonstration on how to perform the behaviour* was coded if the patient was presented with an observable demonstration, but not if only provided with written instructions; this was coded as *instruction on how to perform the behaviour*.

A clinician (RG) extracted data for all included studies. A speech and language therapist (CS) and health psychologist (BG) independently extracted data for four (27%) randomly selected studies. Inter-rater agreement, assessed using Cohen’s kappa, was ‘substantial K = 0.6’ or better for selection of full-text articles assessed for inclusion (K = 0.86), study quality (K = 0.74) and BCTs (K = 0.66) [32].

### Analysis

A meta-analysis was not used due to the small number of studies and the large variability. Furthermore, it would not have been as informative for the purpose of addressing our study questions. Instead we selected a qualitative method that combined the use of summary tables, and qualitative exploration of the data.

We used a synthesis approach [19] to describe and explore our findings. Results are structured and presented in line with the key steps of this approach as listed below:

1. *Developing a theory or model of how the intervention might work*: Our logic model illustrating the interaction of various components of the intervention within a health service system has been presented above.
2. *Preliminary synthesis of the findings* – We summarise the characteristics of the included studies tabulating the same features across all studies. Additionally, we present summary tables of the intervention characteristics (behavioural strategies) extracted from studies and examples of these strategies obtained from content analysis of the study reports.
3. *Exploring relationships in the data* – We present observations of relationships between studies that may explain differences in outcomes and the direction and size of intervention effects. We assumed that BCTs that featured at least twice as frequently in studies that showed a statistically significant positive effect on at least one outcome measure (p < .05) in favour of the intervention group may show some promise, or at least justify more rigorous evaluation.
4. *Assessing the robustness of the synthesis*. We reflect on the number and quality of the studies included, and the methods used in synthesizing the findings.

## Results

### Synthesis of study and intervention characteristics

#### Study selection

Of 374 articles identified from the combined searches, 254 remained after de-duplication. Twenty-nine articles were retained following title and abstract screening, of which 15 studies, each reporting one intervention, were eligible for review. No additional studies were included following the hand-search of reference lists.

#### Study characteristics

The 15 studies were undertaken across seven countries (USA, 7 studies; Netherlands and China, 2 studies respectively; Denmark, Sweden, Austria, Japan, 1 study respectively). All were carried out in a university hospital, medical centre or cancer centre. All studies sought to evaluate the impact of swallowing exercises, on one or more swallow related outcomes. Eight were randomised trials [22, 33–39], and seven were non-randomised controlled trials [40–46]. Six studies reported a comparator group of ‘no treatment’[36–38, 42–44] and two of delayed treatment [40, 45]. In two studies, treatment as usual was described as dietary advice without exercise [33, 34]. The comparator group for the remaining studies used a different swallowing exercise protocol described as usual care for that setting.

Follow-ups took place between one and 12 months. The measure used for baseline swallowing status varied greatly, with 5 studies [40, 42–45] providing no report of swallowing function at baseline. At least 14 different outcome measures relating to swallow function were reported across the studies and at varied time intervals (Additional file 3: Table S3). The most frequently used measures (7/15) were: modified barium swallow and use of a feeding tube as a surrogate marker of swallow (dys) function. The PSS or a patient rated diet texture score, mouth opening, penetration-aspiration scale (PAS) [47], MDADI and weight measures were also used across multiple studies, although less frequently. Almost all studies reported a combination of instrumentally derived (objective), patient-reported and/or clinician rated outcomes measures. Two studies [42, 45] reported on just the MDADI, and one study [46] reported on a diet texture score alone.

#### Sample characteristics

A total of 995 participants were reported at the commencement of the studies (Table 1; 729 males, 257 females, nine unclear). Sample size ranged from 18 to 374. Average age across studies was 59.4 years. Both the gender and age demographics are broadly reflective of the epidemiology of HNC [48, 49].

**Table 1:** Study demographics and sample characteristics

Author/year Country of Origin.	Setting	Type of study	Type of intervention (I) Control (C)	Oncology Treatment, sample characteristics	Sample size T = total I = new treatment, C = control	Gender (M:F)	Sample age for (I) and (C) groups. (mean and SD/range)	Baseline Swallowing status	Length of Follow-up
Mortensen 2015 [33] Denmark	University hospital	RCT (pre-treat)	(I) Individualised dietary advice, exercise protocol of standard exercises – 10reps/3× daily (C) = usual care, individual dietary advice. VFS and advice as needed. (active control)	Cancer of larynx, pharynx, oral cavity (T2-T4), unknown primary. Planned for radiotherapy with/without chemo. No previous oncology treatment.	T = 39 I = 19 C = 20 NB: 5 patients excluded at start.	34:5	I = 58 (39–77) C = 59 (40–74)	(I) SPSS =1.44 (C) SPSS = 1.38	11 months
Van Den Berg 2014 [34] Netherlands	University medical centre	RCT (pre-treat)	(I) = combined diet counseling and individualized swallow therapy. (C) = weekly individual diet counseling for better nutrition. (active control)	Patients with stage II-IV HNC treated with postoperative radiation with/without chemotherapy.	T = 120 I = 60 C = 60	89:31	I = 63 (33–83) C = 60 (40–86)	(I) PSS mean =78 (SD =26) (C) PSS mean =75 (SD = 25)	30 weeks
Ohba 2014 [40] Japan	University hospital	Retro-spective case–control design (peri-treatment)	(I) = shaker exercise during CRT. (C) = Mendelsohn manoeuvre only when dysphagia developed (delayed active)	Advanced HNC, laryngeal, oropharyngeal, hypopharyngeal cancers.	T = 51 I = 21 C = 30	46:5	I = 65 (53–80) C = 63 (49–89)	Not reported	2-4 weeks
Lazarus 2014 [35] USA	Medical centre	RCT (post-treat)	(I) = isometric tongue exercises with traditional exercises. (C) = traditional exercises including ROM. (active control)	Patients with stage II-IV oral and oro-pharyngeal cancer, who previously underwent radiotherapy with/without chemo.	T = 23 I = 12 C = 11	22:1	I = 62.3 (SD, 8.06) C = 61.7 (SD, 7.27)	(I) OPSE mean = 44.63 (dysphagia if less than 39) Tongue strength = 44.63 (C) OPSE =59.6 tongue strength = 49.3	10 weeks
Virani 2013 [41] USA	Cancer centre	Non randomised trial – matched groups. (pre-treat)	(I) = behavioural swallow exercises (C) = repetitive swallowing tasks (active control)	Newly diagnosed HNC of the oral cavity, oropharynx, nasopharynx, larynx or unknown primary due to undergo radiotherapy with/without chemo.	T = 50 I = 26 C = 24	40:10	I = 64 (24–90) C = 60 (43–85)	(I) FOIS =6.5 (C) FOIS =6.6	3 months
Kotz 2012 [35] USA	Academic medical centre	RCT (pre-treat)	(I) = behavioural swallow exercises (5sets) (C) = no active treatment	Patients with HNC receiving CRT, excluding any surgery or previous radiation or previous history of dysphagia.	T = 26 I = 13 C = 13	20:6	I = 57 (SD,10) C = 62 (SD,11)	I) FOIS =7 PSS =100 (C) FOIS =7 PSS =100	12 months
Carnaby-Mann 2012 [37] USA	University Hospital Cancer Centre	RCT 3-arms (pre/peri)	(I) = pharyngocize and diet modification. (C) = usual care consisting of supervision for safe swallowing. Sham therapy – buccal extension manoeuvre –daily schedule. Active control – sham, and no treatment group	Newly diagnosed with oropharyngeal cancer and planned for external beam radiotherapy with/without chemo. TNM stage 1-4	T = 58 I = 20 C = 20 Sham =18	44:14	I =59 (SD,10.4) C = 54 (SD, 11.3) sham = 60 (SD, 12.2)	(I) MASA = 195.1 SD = 5.9 (C) MASA =195.5 SD = 4 sham = 194.7 SD = 3.5 scores >178 suggest no dysphagia.	6 months
Zhen 2012 [42] China	University Hospital	Quasi-experiment- Parallel cluster study (post-treat)	(I) = 30 min swallow training daily for 2 weeks (C) = no active treatment	All patients were post tongue surgery. MDADI score of 60 or lower on screening.	T = 46 I = 23 C = 23	29:17	I =60.52 (SD,5.5) C = 57.5 (SD, 5.72)	Not reported	1 month
Ahlberg 2011 [43] Sweden	University Hospital	Non randomized parallel groups (pre-treat)	(I) = pre-treatment swallowing exercises. (C) = no active pretreatment intervention	Patients diagnosed with HNC due to receive curative radiotherapy	T = 374 I = 190 C = 184	253:121	I =63.6 (SD, 13.1) C = 64.1 (SD, 12)	Not reported	6 month outcomes, 2 year F/U.
Tang 2011 [38] China	University Hospital	RCT (post-treat)	(I) = exercises and jaw stretch (C) = no active exercise intervention	Previously diagnosed with nasopharyngeal cancer and received radiotherapy – long term post-treatment.	T = 46 I = 25 C = 21 3 pts excluded	32:11 (gender of patients excluded not reported)	T =49.3 (not indicated separately for groups)	(I) WST =3.6 IID = 1.89 (C) = WST =3.8 IID =1.8	3 months
Van Der Molen [22] 2011 Netherlands	Cancer Centre	RCT (pre-treat)	(I) device based rehab protocol using therabite (C) standard treatment of best evidence- based exercises (active control)	Stage III-IV HNC (oral cavity, oropharynx, hypopharynx, larynx, nasopharynx) planned for curative chemo-radiation treatment.	T = 55 I = 27 C = 28	39:10 (gender of patients excluded not reported)	I = 56 (37–78) C = 57 (32–75)	Baseline function of each group not reported. Overall mean at pre-treatment: FOIS =7	^*^10 weeks 2 years, 6 years FU in later papers.
Logemann 2009 [39] USA	7 settings university hospitals cancer centres	RCT (post-treat)	(I) shaker exercise (C) traditional swallow therapy (active control)	Patients with prolonged oro-pharyngeal dysphagia of at least 3-month duration	T = 19 I = 8 C = 11	16:3	Not provided	All had aspiration	6 weeks
Carroll 2007 [44] USA	University hospital	2-arm Retrospective Case control Study (pre-treat)	(I) pre-treatment swallowing exercise protocol. C) usual care -swallow rehab as problems arose post treatment. (no active pre-treat exercises)	Patients with advanced squamous cell cancer of the oropharynx, hypopharynx and larynx treated with chemo-radiation.	T = 18 I = 9 C = 9	12:6	I = 57.5 C = 60.7	Not reported	12 months
Kulbersh 2006 [45] USA	University Hospital	2-arm Prospective cohort study (pre-treat)	I) pre-treatment swallowing exercise protocol (C) exercises given at first visit after treatment. (delayed intervention)	All patients diagnosed with HNC with/without nodal disease but without metastatic disease	T = 37 I = 25 C = 12	28:9	I =55.1 (SD, 9.6) C = 66.3 (SD, 10)	Not reported	12 months
Denk 1997 [46] Austria	ENT department	Non-randomised, 2-arm parallel group study (post-treat)	(I) therapy with video-endoscopic biofeedback. (C) conventional swallow therapy. (active control)	Patients with prolonged post-operative aspiration following resection of malignant tumours of the oropharyngeal swallowing structures.	T = 33 I = 19 C = 14	25:8	I = 54 (37–68) C = 53 (37–79)	Prolonged post-op aspiration, with tube feeding	Variable, based on time to establish oral intake

Notes: (I) = intervention group; (C) = control group; T = total sample; RCT = randomised controlled trial; HNC = head and neck cancer; SPSS = swallowing performance status scale; PSS = performance status scale; OPSE = oro-pharyngeal swallow efficiency; FOIS = functional oral intake scale; MASA = Mann swallowing assessment; WST = water swallow test; IID = inter-incisor distance; * Later papers linked to this study include follow-up measures at 2-years, and 6 years

Patients’ HNC diagnosis ranged from stage II to stage IV disease. The sites included the oral cavity, oropharynx, hypopharynx, nasopharynx and larynx. The majority of studies (12/15), focused on the group of patients treated with radiotherapy or chemo-radiation. Of these 12 studies, ten focused on *pre*-*treatment* swallowing interventions. Three of the 15 studies [39, 42, 46] targeted patients who were treated with surgery as the main modality (Table 1).

#### Quality assessment

As indicated in Table 2, only one study [37] achieved a score ≥6 and met the criteria for good quality [24]. In 7/15 studies, there was at least one item for which information was missing or could not be deduced from the study report. Scores ranged from 0–7 out of 11. No study complied with criteria requiring that the *therapist and subject were blinded to the intervention* (15/15) (Table 2).

**Table 2:** Quality assessment ratings for all studies included in the review

	Mortensen	Van Den Berg	Ohba	Lazarus	Virani	Kotz	Carnaby Mann	Zhen	Ahlberg	Tang	Van Der Molen	Logemann	Caroll	Kulbersh	Denk
✓ = yes															
?=															
✗ = no															
Quality criteria															
Randomisation detailed	✓	✓	n/a	✓	n/a	?	✓	n/a	n/a	?	✓	✗	n/a	n/a	n/a
Allocation concealed	?	✗	n/a	?	n/a	✗	✓	n/a	n/a	✗	?	✗	n/a	n/a	n/a
Similar groups at baseline	✓	✓	✓	✓	✓	✓	✓	✓	✓	✓	✓	✓	✓	✗	✓
Subject blind	✗	✗	✗	✗	✗	✗	✗	✗	✗	✗	✗	✗	✗	✗	✗
Therapist blind	✗	✗	✗	✗	✗	✗	✗	✗	✗	✗	✗	✗	✗	✗	✗
Assessor blind	?	✗	✗	✓	✓	✓	✓	✗	✗	✗	✗	✓	✓	✗	✗
Co-intervention controlled	?	✗	✗	?	✗	?	✓	✗	✗	✗	?	?	?	✗	?
Acceptable compliance	✗	?	?	✗	✗	✗	✗	✓	?	✓	?	?	?	?	?
Acceptable withdrawal rate	✗	✓	✓	✗	✓	✗	✗	✓	✗	✓	✓	✗	✓	?	✓
Timing of outcome	✓	✓	✓	✓	✓	✓	✓	✗	✓	✓	✓	✓	✓	✗	✗
Intention to treat	✓	✓	✗	?	✓	✓	✓	✗	✗	✗	✓	✓	✗	✗	✗
TOTAL	4	5	3	4	5	4	7	3	2	4	5	4	4	0	2

### Intervention characteristics

Twenty individual BCTs (Table 3) were each identified in at least one intervention. The average number of BCTs per intervention was seven, with a range of four to ten. The BCT *instruction on how to perform the behaviour* was reported in all interventions (15/15), with 14/15 including *setting behavioural goals* (for example, perform jaw exercises 3×/day) and 13/15 including *action planning* (for example perform exercises before mealtimes) (Additional file 1: Table S1).

**Table 3:** Behaviour Change Techniques (BCTs) identified across included studies

Actual check ticks (✔) = BCT present	Mortensen	V.D. Berg	Ohba	Lazarus	Virani	Kotz	Carnaby	Zhen	Ahlberg	Tang	V.D. Molen	Logemann	Carroll	Kulbersh	Denk	% studies
Goals and Planning																
Goal setting (behaviour)	✓	✓	✓	✓	✓	✓	✓		✓	✓	✓	✓	✓	✓	✓	93
Problem solving	✓															7
Action planning	✓	✓	✓	✓	✓	✓	✓		✓	✓	✓	✓	✓	✓		87
Review behaviour goals							✓		✓							13
Review outcome goals															✓	7
Feedback and Monitoring																
Monitoring of behaviour by others without feedback		✓		✓										✓		20
Feedback on behaviour						✓									✓	13
Self monitoring of behaviour	✓			✓		✓	✓			✓		✓				40
Monitoring outcome of behaviour without feedback									✓							7
biofeedback															✓	7
Social Support																
Social support (unspecified)		✓		✓	✓	✓		✓	✓	✓						47
Social support (practical)	✓						✓			✓		✓			✓	33
Shaping Knowledge																
Instruction on how to perform the behaviour	✓	✓	✓	✓	✓	✓	✓	✓	✓	✓	✓	✓	✓	✓	✓	100
Comparison of behaviour																
Demonstration of the behaviour							✓									7
Associations																
Prompts and cues						✓										7
Repetition and Substitution																
Behavioural practice/rehearsal		✓	✓	✓	✓	✓	✓	✓		✓	✓	✓	✓	✓		80
Habit formation											✓		✓			13
Generalization of target behaviour	✓	✓			✓	✓				✓	✓	✓	✓			53
Comparison of outcomes																
Credible source	✓	✓				✓	✓	✓	✓	✓		✓		✓		60
Antecedents																
Adding objects to the environment				✓			✓		✓	✓	✓		✓			40
Total Number of BCTs	8	8	4	8	6	10	10	4	8	10	7	8	7	6	6	

A total of three Function categories were each identified in at least one intervention. *Training* was identified in all interventions (15/15), *Education* in 12/15 and *Enablement* for example providing patient with a TheraBite device in 5/15 (Additional file 2: Table S2).

Regular performance of the prescribed swallowing exercises was the target behaviour for all interventions. Due to the small number of studies, and the variation in exercise content we made no attempt to further group interventions according to the exercise type (Table 3).

### Exploring relationships between behavioural strategies and effectiveness

#### Frequency of behavioral intervention components and intervention effectiveness

The three most commonly used BCTs that appeared in > 85% of interventions were *instruction on how to perform the behaviour*, *setting behavioural goals* and *action planning*. These BCTs may arguably form the cornerstone of exercise therapy interventions so it is unsurprising that they were identified in >85% of interventions. Four BCTs were used in at least twice as many interventions that produced positive effects relative to those with no such effects - *practical social support*, *behavioural practice*/*rehearsal*, *self*-*monitoring*, and *credible source*.

### Exploring relationships between trial methods and effectiveness

#### Influence of comparator group on intervention effectiveness

We wished to explore any relations between active and non-active comparator groups and intervention effectiveness. Of five studies [22, 33–35, 43] reporting no evidence of a significantly positive effect of the intervention on any outcome, four had an active control group where similar behavioural strategies were used in both the intervention and comparator groups, except Ahlberg [43] who used parallel groups on different sites. The active comparator group represented either a different exercise regime (often described as usual care), or may have omitted the use of a swallowing exercise device that was included in the intervention group.

Of the ten interventions that demonstrated evidence of positive effects on at least one swallowing outcome measure (Additional file 3: Table S3), five [36–38, 42, 44] had a *non*-*active comparator* group. In two studies [40, 45], intervention was delayed and therefore effectively represents a non-active comparator group. Two studies had an active comparator group that received a different exercise intervention [39, 41]. One study [46] used similar exercise interventions but the intervention group included *biofeedback* by providing the patient with visual feedback of swallowing during a fibreoptic endoscopic assessment. One study [37] had 3 groups: a treatment group receiving swallowing exercises, a group receiving sham exercises using a similar dose schedule and a usual care group who received only safe-feeding advice by the hospital team when required but not an exercise intervention. The authors found a statistically significant difference between each of the active groups (swallowing exercises and sham exercises) and the usual care group, but a smaller difference (favouring the exercise group) between the swallowing exercise group vs sham exercise group.

Again we acknowledge the small number of studies, however our findings seem to indicate that employing active comparator groups particularly when similar behavioural strategies are used, are less likely to demonstrate statistically significant positive effects. Interestingly, a positive effect was still found in one study [46] when both groups received similar exercise interventions, but different non-exercise content (intervention group received biofeedback, a named BCT).

#### Type and timing of outcome measures and intervention effectiveness

Outcomes that significantly improved with the exercise intervention did so mostly at 1 month post oncological treatment, with a general decline in effect at the later time-points after treatment. Four studies measured outcomes at 12 months [33, 36, 44, 45] but only one [45] showed a significant difference in favour of the intervention by this time-point. In one study [36], outcomes were measured at multiple time-points; significant differences were observed at 3 and 6 months post-treatment but not at 9 and 12 months (Additional file 3: Table S3). Another study [33] charted a rapid decline in patient adherence to swallowing exercises over the first 12 months following treatment.

Outcomes broadly classified as *objective measures* (PAS, MBS score, mouth opening, feeding tube) were more frequently improved by the intervention, when compared to patient reported and clinician rated measures.

This exploration of the data has highlighted the potential impact that BCTs and trial methods such as choice of comparator group and timing of outcome measures may have on intervention effectiveness. Implications of these findings are expanded upon in the Discussion.

## Discussion

We identified 15 controlled clinical trials (8 randomised) that currently represent the best available evidence of swallowing interventions for patients with HNC, and extracted three function categories and 20 different BCTs that characterize these interventions. By specifically isolating these BCTs, we may encourage more consistent descriptions of the non-exercise content of swallowing exercise interventions in the literature increasing our ability to replicate studies more accurately. Indeed, in time it may be possible to devise interventions that test the effectiveness of specific BCTs or groups of BCTs used in swallowing exercise interventions for this patient population, and to link these to underlying theory and mechanisms of change [50]. In so doing, we may be better placed to understand why interventions work, for whom and in which contexts [11].

We also examined the data for any relationships that may elucidate the interaction of different components of this complex intervention. For example, studies that employed active comparator groups using similar BCTs to the intervention group were more likely to demonstrate non-significant results. Furthermore in a trial that employed three groups [37], (an exercise group, a sham exercise group, and a non-active control group), the authors reported that the active sham exercise group that received similar BCTs to the pharyngocize (exercise treatment) group achieved much better outcomes compared to the non-active control group. It may therefore be the constituent BCTs that were responsible for intervention effectiveness, by stimulating greater adherence to the prescribed treatment. Whilst the authors themselves did not specifically make reference to BCTs, they did question whether the ‘benefits obtained from the sham group could be ascribed to the placebo effect of behavioral attention’ (p.219). Equally they speculated that the sham exercise (done diligently) might have had an intrinsic benefit from the increased movement of oral musculature. Regardless, these findings raise the possibility that BCTs may be functioning as *active ingredients* influencing intervention outcomes. For most studies where both the intervention and active comparison group used similar BCTs, no statistical significance in outcomes between groups was reported. This might be because the interventions given to both groups were too similar, or because of a lack of power due to small sample sizes. However it does raise other interesting questions: What contribution do BCTs add to intervention outcomes, and how does their presence in usual care/placebo interventions impact effectiveness? Reporting of swallowing exercise interventions tends to focus mainly on the treatment group and often provides only cursory reference to the usual care group. The findings of this review highlight that the same methodological care should be taken in devising the treatment manuals for the intervention and comparator groups ensuring that behaviour change components are also specified, given their potential to impact patient adherence and subsequent outcomes. This may prevent hasty conclusions that imply swallowing exercises have no benefit, rather than the conclusion that the “new intervention” was not shown to demonstrate any significant additional benefit over usual care.

The variability in the type and time-point of the primary outcome measures for clinical trials in this field restricts the ability to satisfactorily pool data or compute effect sizes to address the efficacy of swallowing interventions in patients with head and neck cancer. We generally observed that in studies that reported a positive outcome, this was mostly seen in the short term. One reason for this may be because patients do not continue with their exercises long term. Behavioural strategies such as *habit formation*, requires that an individual repeatedly perform the behaviour in the same context such that it becomes automatic. This automaticity may promote maintenance of exercises as it may over-ride conscious intentions [51] and could have a role to play in improving swallowing outcomes longer term. We also observed that outcomes collected after 6 months showed little difference between groups. This was especially relevant for patient-reported outcomes that arguably may also reflect patients’ changing expectations and adaptation over time, and not just functional swallowing status. Furthermore, this mirrors the usual trajectory of behaviour change where short-term goals are given priority. Rothman [52] highlighted that the psychological factors that underpin initiation of a new behaviour differ from those that predict maintenance of the behaviour. By implication, different BCTs may be required for these distinct phases. It was also noted that few studies actually collected objective measures of swallowing in the longer term, making it difficult to assess changes in swallow physiology at later time-points. Standardizing outcome measures and agreement on the key evaluation time-points will greatly progress efforts to understand if swallowing exercise interventions are indeed beneficial for this group of patients and over what time period. Consideration should also be given to the expected trajectory of swallowing recovery after head and neck cancer treatment including the possible onset of late effects of treatment such as post radiation fibrosis known to impair swallow function [53, 54].

### Assessing the robustness of the synthesis

According to Popay and colleagues [19], robustness of a synthesis is usually determined by 1) the methodological quality of the included studies, 2) methods used to minimise bias in the synthesis process, and 3) whether detailed information has been provided on the type of studies included/excluded. This review meets the latter two criteria by providing detailed information *via* a published protocol. Methodological quality of the available evidence was rated as poor with only one study meeting more than 50% of the applied quality criteria. It is however acknowledged that for this type of intervention, it is usually impossible to blind the therapist and subject to the intervention. Attrition is a common feature for studies that involve a complex intervention within a multifaceted cancer care pathway, and randomised studies within this field are only beginning to emerge [16, 25]. Excluding studies that did not meet quality criteria may therefore have disadvantaged our ability to address our primary aims in this exercise. Furthermore, complex interventions may require a differing emphasis on the markers of study quality as they are frequently evaluated within the context of pragmatic clinical trials. Since developing our protocol, new methods of evaluating quality in complex interventions have begun to emerge that may be more suitable for future use [9].

### Limitations and challenges

This review is limited by the fact that the accuracy of the coding scheme relies on the quality of published intervention reports, which are often not sufficiently detailed to extract all necessary components of the intervention [55]. It is possible therefore that the intervention itself may have included strategies that have not been coded in this review. Descriptions of the treatment delivered to comparator groups in particular were poor, and in some cases decisions about the presence of BCTs in the comparator group had to be based on the authors’ implicit suggestions that interventions were identical apart from the specific exercise protocol used in each of the active groups.

Despite the BCT taxonomy being developed within Behavioural Science, there is ongoing debate amongst experts in behaviour change as to its merits. Critics have questioned the value of coding BCTs, suggesting it creates a level of abstraction that detracts from the detailed content analysis of interventions [56]. As a counter argument, we believe that in a clinical field that has focused mainly on exercise protocol content, drawing attention to broader more abstract process based mechanisms can only enrich our understanding of complex interventions. The taxonomy brings structure, organization and a common language to this process. For example, coding a BCT such as *self*-*monitoring* may not tell us how the self-monitoring was done, but it does highlight that the use of self-monitoring may be relevant to changing adherence behaviour, particularly when it is frequently observed in successful interventions.

### What this review adds

This review applied a behavior change perspective to studies within head and neck cancer swallowing rehabilitation, with a specific focus on identifying the behavioral strategies that may impact patient adherence to exercises, and consequently swallowing outcomes. Such an analysis is absent in the current literature. Our aim was to instigate discussion and greater thought about the complexity of swallowing exercise interventions, their design and the reporting of such interventions. It addresses the question of *what* might bring about change by isolating the specific components within an intervention, other than the nature of the treatments to which patients are encouraged to adhere, that may influence behaviour [57]. It therefore expands on the findings from previous related reviews [15, 16, 58, 59] and goes some way to highlighting additional components that may be present and active in this complex intervention. Given the relative paucity of high quality data, the review did not attempt to definitively answer the question of which BCTs are most effective in promoting adherence, but instead aimed to highlight those that were prevalent in successful interventions. Using this as a starting point, we may begin to design future interventions incorporating specific BCTs or groups of BCTs to examine more closely whether they strengthen interventions aimed at improving swallowing function *via* swallowing exercises. Clearly BCTs are only one part of trial design and equal attention should be placed on other important aspects such as precise definition of the whole intervention package in prospective study protocols and intervention manuals.

This approach seeks to generate new discussion toward understanding the make-up of complex interventions. It also offers new perspectives in the interpretation of findings from clinical trials of swallowing exercises where it is clear that evaluating effectiveness is hampered by poor adherence.

## Conclusion

The effectiveness of swallowing exercises depends in part on adherence to exercises. This review looks at BCTs – these seem to promote adherence. The review has provided preliminary information about which BCTs occur in reports of complex swallowing interventions and has highlighted that behavioural components may be *active ingredients* of change that impact intervention outcomes. It is likely that many BCTs are used in clinical practice, and there will be some bias towards the techniques that researchers tend to report. Nevertheless, introducing the taxonomy of BCTs helps equip dysphagia researchers with the tools and the language to improve consistency in how complex interventions are specified in research protocols, intervention manuals and the published literature study. In time, the approach can also be used in examining fidelity in the delivery of interventions through field testing and observational methods. Its merits and weaknesses can only be adequately evaluated as the body of work adopting this approach increases.

## Additional files

Additional file 1: Table S1. BCTs, descriptions and examples from studies included in the review. NB: A complete list of BCTs in the taxonomy (BCTTv1) and a full description can be found in Michie, Atkins & West [60]. (DOCX 125 kb)
Additional file 2: Table S2. Intervention Function definitions and examples from included studies where identified. NB: Further general examples of Intervention Functions can be found in Michie, Atkins & West [60]. (DOCX 82 kb)
Addtional file 3: Table S3. Outcome measures obtained at four time points post oncology treatment. (DOCX 1082 kb)

## Abbreviations

BCT: Behaviour change technique
BCTTv1: Behaviour change technique taxonomy Version 1
EORTC: European Organisation of Research and Treatment of Cancer
FOIS: Functional oral intake scale
HNC: Head and neck cancer
MBS: Modified barium swallow
MDADI: MD Anderson Dysphagia Inventory
PAS: Penetration-aspiration scale
PRISMA: Preferred Reporting of Items for Systematic reviews and Meta Analyses
PSS: Performance status scale
QOL: Quality of life
WST: Water swallow test
