# Supplementary material for: Swallowing interventions for the treatment of dysphagia after head and neck cancer: a systematic review of behavioural strategies used to promote patient adherence to swallowing exercises
Source: BMC Cancer. 2017 Jan 10;17:43. doi: 10.1186/s12885-016-2990-x (PMC5223405; doi:10.1186/s12885-016-2990-x)
Supplement: Additional file 1: Table S1. — BCTs, descriptions and examples from studies included in the review. NB: A complete list of BCTs in the taxonomy (BCTTv1) and a full description can be found in Michie, Atkins & West [60]. (DOCX 125 kb) [file 12885_2016_2990_MOESM1_ESM.docx]

**Table A1. BCTs, descriptions and examples from studies included in the review**

APPENDIX A

| BCT | Description (taken from BCTTv1) | Example/s |
| --- | --- | --- |
| Goal setting (behaviour) | Set or agree a goal in terms of the behaviour to be achieved.  NB: if goal also defines a specific context,frequency, duration or intensity – also code *Action planning.* | Participants were instructed to perform these exercises for 10 repetitions, 5 times a day (Carroll, 2007).  Intervention patients were asked to perform 3 sets of 10 repetitions of each exercise on a daily basis (Kotz, 2012). |
| Problem solving | Analyse or prompt the person to analyse factors influencing the behaviour and generate or select strategies that include overcoming barriers/increasing facilitators (includes *relapse prevention* and *coping planning*). | If acute side effects restricted the performance of the exercises, patients were instructed to accomplish as much as possible and to resume to the complete exercise program whenever possible. Patients were encouraged to continue oral food intake if in any way possible and safe (Mortensen, 2015). |
| Action planning | Prompt detailed planning of performance of the behaviour (must include at least one of context, frequency, duration, intensity). Context may be environmental (physical or social) or internal (physical, emotional, or cognitive- includes *implementation intentions.*) | The Shaker exercise consisted of three 1-min head lifts in the supine position with a 1-min rest between lifts [5]. These sustained head-raising exercises were followed by 30 consecutive repetitions of head raisings in the same supine position. For both sustained and repetitive head raising, volunteers were instructed to raise the head high enough to be able to observe their toes without raising their shoulders (Logemann,2009).  Using a 3-times-a-day paradigm, an individual could coordinate the exercises with either breakfast, lunch, and dinner or morning, noon, and night to promote better recall (Kotz, 2012) |
| Review behaviour goals | Review behaviour goals jointly with the person and consider modifying goals or behaviour change strategy in light of achievement. This may lead to re-setting the same goal, a small change or setting a new goal or in addition to first, or no change. | At follow-ups, the directions could sometimes be changed to a ‘hold and release’ technique, depending on the need and/or compliance of the patient (Ahlberg, 2011). |
| Review outcome goals | Review outcome jointly with person and consider modifying goals in light of achievement. | The outcomes after discharge was recorded by the patient or caregiver in a diary and reviewed at monthly telephone interviews (Carnaby-Mann,2012) |
| Monitoring of behaviour by others without feedback | Observe or record behaviour with the person’s knowledge as part of a behaviour change strategy. | 25 patients were started on a protocol of swallowing exercises 2 weeks prior to the start of radiation and returned to the clinic at 2 weeks and 6 weeks into their radiation treatment to monitor progress and compliance with the protocol (Kulbersh,2006). |
| Feedback on behaviour | Monitor and provide informative or evaluative feedback on performance of the behaviour (eg. Form, frequency, duration, intensity). | Intervention patients participated in weekly face-to-face swal- lowing therapy sessions with the same head and neck speech pathologist (T.K.), who ascertained the patients’ compliance with the swallowing exercises and reinforced learning to ensure proper technique (Kotz, 2012) |
| Self monitoring of behaviour | Establish a method for the person to monitor and record their behaviour(s) as part of a behaviour change strategy. | Patients were instructed to keep a log oftheir daily performance to further encourage adherence to the swallowing exercise protocol (Kotz, 2012) |
| Monitoring of outcome of behaviour without feedback | Observe or record outcomes of behaviour with the person’s knowledge as part of a behaviour change strategy. | At the clinical screening of swallowing, the patient was asked to complete one swallow of two bolus sizes (5 and 15 ml) of four consistencies: thin liquid, thick liquid, paste, and cookie. Movement ofthe ﬂoor ofthe mouth, hyoid, and thyroid cartilage was evaluated by manual palpation during the act of swallowing. The following swallowing parameters were clinically evaluated: oral manipulation and transport ofbolus, presence of aspiration, laryngeal elevation, need for several swallows, delayed initiation of swallowing, and nasal regurgitation. Aspirations were noted as cough, need to clear the throat, wet voice, or sudden breathing difﬁculties (Ahlberg, 2011) |
| Biofeedback | Provide feedback about the body (eg. Physiological or biochemical state) using an external monitoring device as part of a behaviour change strategy. | 19 patients received treatment with video-endoscopic biofeedback (Denk, 1997). |
| Social support (unspecified) | Advise on, arrange or provide social support (eg. From friends, relatives, colleagues, or staff) or non-contingent praise or reward for performance of behaviour. It includes encouragement and counseling, but only when it is directed at the behaviour. | Each patient had to appoint a guardian who was responsible to oversee the patient’s everyday training (Tang, 2011).  Each patient in both the exercise and swallow treatment groups attended swallowing therapy sessions once every week for 45 minutes throughout the time they were undergoing RT/chemotherapy (Virani, 2013) |
| Social support (practical) | Advise on, arrange or provide *practical* help for performance of behaviour. (arranging pain control to enable continuation of exercises.) | The principal investigator contacted each subject weekly by phone to document compliance with the exercise programmes, as well as too determine pain levels and assess the need for medications per the pain-related questions on the HNCI (Lazarus, 2014) |
| Instruction on how to perform the behaviour | Advise or agree on how to perform the behaviour (includes *skills training*) | When training in the Mendelsohn manoeuvre, subjects were provided verbal, visual, and tactile cues, as well as written instructions (Lazarus, 2014). |
| Demonstration of the behaviour | Provide an observable sample of the performance of the behaviour directly in person or indirectly (eg via film, pictures, for the person to aspire to or imitate), includes *modeling*. | Standardized high-intensity swallowing therapy (“pharyngocise”) included a battery of exercises (e.g., falsetto, tongue press, hard swallow, and jaw resistance/strengthening using the Therabite Jaw Motion Rehabilitation System) and dietary modiﬁcation, under the direction of the study speech pathologist, twice daily for the duration of the CRT (Carnaby Mann, 2012). |
| Prompts and cues | Introduce or define environmental stimulus with the purpose of prompting or cueing the behaviour. The prompt or cue would normally occur at the time or place of performance. | Using a 3-times-a-day paradigm, an individual could coordinate the exercises with either breakfast, lunch, and dinner or morning, noon, and night to promote better recall (Kotz, 2012). |
| Behavioural practice/rehearsal | Prompt practice or rehearsal of the performance of the behaviour one or more times in a context or at a time when the performance may not be necessary, in order to increase habit and skill. | Subjects were trained until they could independently demonstrate the exercises during the baseline training session (Lazarus, 2014)  The rehabilitation training exercises were performed 3 times per day. Each exercise was repeatedly practiced for 15 cycles at each time for a total of 45 cycles per day (Tang, 2011). |
| Habit formation | Prompt rehearsal and repetition of the behaviour in the same context repeatedly so that the context elicits the behaviour. | Participants were encouraged to integrate the exercise into other daily activities such as brushing teeth (Van der Molen, 2011) |
| Generalization of target behaviour | Advise to perform the wanted behaviour, which is already performed in a particular situation, in another situation. | The exercises were trained with the patient and written and drawn instructions were given for doing the exercises at home (Van Den Berg, 2014 |
| Credible source | Present verbal or visual communication from a credible source in favour of or against the behaviour. (code this BCT if source generally agreed as credible (eg. Health professionals, words used to indicate expertise or leader in field) and if the communication has the aim of persuading. | Patients were seen by an experienced and dedicated occupational therapist (the profession undertaking dysphagia therapy in Denmark (Mortensen, 2015).  Intervention patients participated in weekly face-to-face swal- lowing therapy sessions with the same head and neck speech pathologist (T.K.), who ascertained the patients’ compliance with the swallowing exercises and reinforced learning to ensure proper technique (Kotz, 2012). |
| Adding objects to the environment | Add objects to the environment in order to facilitate performance of the behaviour. | The stretch exercise of the E rehabilitation consisted of a passive and slow opening of the mouth using the TheraBite device (Van Der Molen, 2011). |

NB: A complete list of BCTs in the taxonomy (BCTTv1) and a full description can be found in Michie, Atkins & West [57]
