# Supplementary material for: Swallowing interventions for the treatment of dysphagia after head and neck cancer: a systematic review of behavioural strategies used to promote patient adherence to swallowing exercises
Source: BMC Cancer. 2017 Jan 10;17:43. doi: 10.1186/s12885-016-2990-x (PMC5223405; doi:10.1186/s12885-016-2990-x)
Supplement: Additional file 2: Table S2. — Intervention Function definitions and examples from included studies where identified. NB: Further general examples of Intervention Functions can be found in Michie, Atkins & West [60]. (DOCX 82 kb) [file 12885_2016_2990_MOESM2_ESM.docx]

**Table A2: Intervention Function definitions and examples from included studies where identified.**

APPENDIX B

| Intervention Function | Definition | Example |
| --- | --- | --- |
| Education | Increasing knowledge or understanding | The importance and necessity of the exercises were emphasized to the patients and the guardians. (Tang, 2011)  All patients received education about normal and disordered swallowing and had ample time to ask questions. (Van Den Berg, 2014) |
| Persuasion | Using communication to induce positive or negative feelings or stimulate action. |  |
| Incentivisation | Creating an expectation of reward. |  |
| Coercion | Creating an expectation of punishment or cost |  |
| Training | Imparting skills | The patients were instructed, both verbally and with written information, on how to perform mobility exercises for the tongue and larynx (Mendelson’s maneuver). (Ahlberg, 2011) |
| Restriction | Using rules to reduce the opportunity to engage in the target behaviour (or to increase the target behaviour by reducing the opportunity to engage in competing behaviours) |  |
| Environmental Restructuring | Changing the physical or social context |  |
| Modelling | Providing an example for people to aspire to or imitate |  |
| Enablement | Increasing means/reducing barriers to increase capability (beyond education and training) or opportunity beyond environmental re-structuring. | E rehabilitation consisted of a passive and slow opening of the mouth using the TheraBite device. (Van Der Molen, 2011) |

NB: Further general examples of Intervention Functions can be found in Michie, Atkins & West [57]
