# Supplementary material for: Swallowing interventions for the treatment of dysphagia after head and neck cancer: a systematic review of behavioural strategies used to promote patient adherence to swallowing exercises
Source: BMC Cancer. 2017 Jan 10;17:43. doi: 10.1186/s12885-016-2990-x (PMC5223405; doi:10.1186/s12885-016-2990-x)
Supplement: Addtional file 3: Table S3. — Outcome measures obtained at four time points post oncology treatment. (DOCX 1082 kb) [file 12885_2016_2990_MOESM3_ESM.docx]

APPENDIX C

Outcome measures obtained at four time-points after oncology treatment.
